# *Plasmodium* infection suppresses colon cancer growth by inhibiting proliferation and promoting apoptosis associated with disrupting mitochondrial biogenesis and mitophagy in mice

**DOI:** 10.1186/s13071-022-05291-x

**Published:** 2022-06-06

**Authors:** Xin Yao, Yujie Cao, Li Lu, Yuanxia Xu, Hao Chen, Chuanqi Liu, Dianyi Chen, Kexue Wang, Jingxiang Xu, Runqi Fang, Hui Xia, Jiangyan Li, Qiang Fang, Zhiyong Tao

**Affiliations:** 1 Department of Microbiology and Parasitology, Bengbu Medical College, Bengbu, China; 2 Anhui Key Laboratory of Infection and Immunity, Bengbu Medical College, Bengbu, China; 3 School of Fundamental Sciences, Bengbu Medical College, Bengbu, China; 4 Clinical Medical Department, Bengbu Medical College, Bengbu, China; 5 School of Life Sciences, Bengbu Medical College, Bengbu, China; 6 Department of Clinical Laboratory, The First Affiliated Hospital of Bengbu Medical College, Bengbu, China

**Keywords:** *Plasmodium*, Colon cancer, Mitochondrial apoptosis, Mitophagy, Mitochondrial biogenesis

## Abstract

**Background:**

Colon cancer is a common gastrointestinal tumor with a poor prognosis, and thus new therapeutic strategies are urgently needed. The antitumor effect of *Plasmodium* infection has been reported in some murine models, but it is not clear whether it has an anti-colon cancer effect. In this study, we investigated the anti-colon cancer effect of *Plasmodium* infection and its related mechanisms using a mouse model of colon cancer.

**Methods:**

An experimental model was established by intraperitoneal injection of *Plasmodium yoelii* 17XNL-infected erythrocytes into mice with colon cancer. The size of tumors was observed dynamically in mice, and the expression of Ki67 detected by immunohistochemistry was used to analyze tumor cell proliferation. Apoptosis was assessed by terminal deoxynucleotidyl transferase (TdT) dUTP nick-end labeling (TUNEL) staining, and the expression of apoptosis-related proteins including Bax, Bcl-2, caspase-9, and cleaved caspase-3 was detected by western blot and immunohistochemistry, respectively. Transmission electron microscopy (TEM) was used to observe the ultrastructural change in colon cancer cells, and the expression of mitochondrial biogenesis correlative central protein, PGC-1α, and mitophagy relevant crucial proteins, PINK1/Parkin, were detected by western blot.

**Results:**

We found that *Plasmodium* infection reduced the weight and size of tumors and decreased the expression of Ki67 in colon cancer-bearing mice. Furthermore, *Plasmodium* infection promoted mitochondria-mediated apoptosis in colon cancer cells, as evidenced by the increased proportion of TUNEL-positive cells, the upregulated expression of Bax, caspase-9, and cleaved caspase-3 proteins, and the downregulated expression of Bcl-2 protein. In colon cancer cells, we found destroyed cell nuclei, swollen mitochondria, missing cristae, and a decreased number of autolysosomes. In addition, *Plasmodium* infection disturbed mitochondrial biogenesis and mitophagy through the reduced expression of PGC-1α, PINK1, and Parkin proteins in colon cancer cells.

**Conclusions:**

*Plasmodium* infection can play an anti-colon cancer role in mice by inhibiting proliferation and promoting mitochondria-mediated apoptosis in colon cancer cells, which may relate to mitochondrial biogenesis and mitophagy.

**Graphical Abstract:**

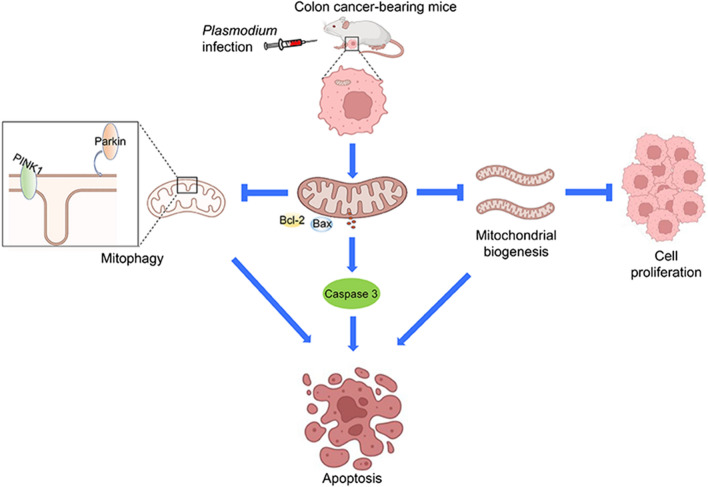

## Background

Colon cancer is one of the most frequent digestive system tumors, ranking fifth in new cases and fatalities among all malignancies worldwide [1]. At present, although surgical resection, radiotherapy, and conventional chemotherapy are used routinely in the treatment of colon cancer [2], the therapeutic effect is still not satisfactory, and new therapeutic strategies are urgently needed.

As early as 1917, *Plasmodium*, one of the most important parasites, was used to treat diseases such as advanced syphilis [3]. Since then, the relationship between *Plasmodium* and cancer has also received attention. According to a report, a negative correlation was found between the incidence of malaria and the mortality rate of some cancers globally from 1955 to 2008 [4]. The antitumor effect of *Plasmodium* infection has been reported in animal studies, including lung cancer [5], hepatocellular carcinoma [6], leukemia [7], and melanoma [8]. However, it has not been reported whether *Plasmodium* infection can inhibit colon cancer.

Past studies on the antitumor mechanism of *Plasmodium* infection have focused on the host antitumor immune response [5], targeting the tumor microenvironment [9] and inhibiting tumor angiogenesis [10]. However, what happens to tumor cells after the host is infected with *Plasmodium* does not get enough attention. It is well known that infinite proliferation and resistance to cell apoptosis in tumor cells are the key to tumor growth [11]. Many therapies exert anticancer effects by promoting tumor cell apoptosis, especially mitochondria-mediated apoptosis [12–14]. Parasites such as *Toxoplasma gondii* [15, 16] exert an antitumor function by inducing apoptosis. However, it is not clear whether *Plasmodium* infection can also play an antitumor role by inhibiting proliferation and inducing apoptosis.

An increasing number of studies have shown that there is a close link between mitochondria and cancer development [17, 18]. Mitochondria are fundamental for cell growth and proliferation in cancer, and metabolic imbalances or increased resistance to mitochondrial apoptosis are prominent features of cancer cells [19]. Indeed, mitochondria maintain cellular homeostasis by regulating mitochondrial biogenesis and mitophagy [20, 21]. Mitochondrial biogenesis can generate new functional mitochondria to increase the number of mitochondria through transcriptional regulation [22], and inhibition of mitochondrial biogenesis can inhibit tumor cell proliferation [23]. Mitophagy is a form of autophagy by which the damaged or superfluous mitochondria are phagocytosed and degraded [24–26], and inhibition of mitophagy can aggravate mitochondrial damage and promote mitochondria-mediated apoptosis [27, 28]. Because mitochondria play a key role in cell proliferation and death, many studies have demonstrated that targeting mitochondria can inhibit tumor growth or induce apoptosis [29, 30]. However, whether mitochondrial biogenesis or mitophagy is involved in the antitumor effect of *Plasmodium* infection requires further exploration.

In this study, we used a murine colon cancer model to investigate the anti-colon cancer effect of *Plasmodium yoelii* infection and its potential mechanism. Our results suggest that *Plasmodium* infection can inhibit tumor growth in mice by suppressing proliferation and inducing apoptosis, which may relate to the inhibition of mitochondrial biogenesis and mitophagy in colon cancer cells. These results will contribute to developing novel strategies for colon cancer treatment.

## Methods

### Laboratory animals, cells, and parasites

BALB/c mice (female, 6–8 weeks old and 18–20 g) were purchased from Changzhou Cavens Laboratory Animal Co., Ltd. and were maintained under a controlled temperature of 20–25 °C and relative humidity of 40–50%. All experiments were reviewed and approved by the Experimental Animal Management and Ethics Committee of Bengbu Medical College, Bengbu, China (approval no. 2021-322).

The nonlethal *P. yoelii* 17XNL strain was donated by Professor Yaming Cao of China Medical University and preserved in our laboratory.

The CT26.WT mouse colon cancer cell line was purchased from Guangzhou Cellcook Biotech Co., Ltd. and cultured in Roswell Park Memorial Institute (RPMI) 1640 medium (Gibco, USA) containing 10% fetal bovine serum (FBS) and 1% penicillin–streptomycin (100 units/ml penicillin and 100 µg/ml streptomycin) under 37 °C and 5% CO_2_ in an incubator.

### Establishment of the murine colon cancer model and infection with *P. yoelii*

A colon cancer model was established as described by Kawakubo et al. [31]. Ten BALB/c mice were subcutaneously inoculated with 0.1 ml CT26.WT cell suspension (5 × 10^6^/ml) per mouse below the axilla of the right forelimb. On the day of tumor formation (on the sixth day after tumor cell injection), the 10 mice were randomly divided into two groups (five animals per group). In the CT26.WT + P.y group, as the *P. yoelii*-infected group, each mouse was intraperitoneally injected with 1 × 10^6^ *P. yoelii*-infected erythrocytes. The mice in the control group (CT26.WT) were intraperitoneally injected with 1 × 10^6^ normal erythrocytes. Tumor growth was observed every third day from the sixth day following inoculation with colon cancer cells. In addition, the levels of parasitemia were observed and counted every third day from the day of *Plasmodium* injection.

### The observation of tumor growth

When the tumors were measurable in tumor-bearing mice (on the sixth day after tumor inoculation), the long diameter *a* (mm) and short diameter *b* (mm) of the tumor were measured with a vernier caliper every third day, and the tumor volume was calculated according to the formula *V* = (*ab*^2^)/2. On the 24th day after tumor inoculation, the tumor-bearing mice were euthanized, and tumors were harvested, weighed, and photographed for further analysis.

### Immunohistochemical staining 

The tumor specimens were fixed with 4% paraformaldehyde solution for paraffin embedding and sectioning. After xylene dewaxing, gradient ethanol hydration, and antigen high-pressure repair, tumor samples were stained with primary antibody (Abcam, USA; Cell Signaling Technology, USA; diluted as follows: 1:400 for Ki67 and Bax, 1:500 for Bcl-2, 1:300 for caspase-9 and caspase-3, 1:2000 for cleaved caspase-3) and incubated at 37 °C for 1 h. The secondary antibody was cultivated at 37 °C for 30 min. After DAB staining and observation under a high-magnification microscope (×400), the positive results were brown in color. Image-Pro Plus 6.0 software was used for statistical analysis, and the average optical density value was used to reflect the protein expression level.

### TUNEL staining

The tumor tissue specimens were fixed with 4% paraformaldehyde and sectioned with paraffin embedding. Terminal deoxynucleotidyl transferase (TdT) dUTP nick-end labeling (TUNEL) assay was performed using the One-Step TUNEL Apoptosis Assay Kit, DAPI Staining Solution, and Antifade Mounting Medium (Beyotime Biotechnology), according to the manufacturer’s protocol. Under a fluorescence microscope (×400), the normal cell nuclei were stained blue and the apoptotic cell nuclei were stained green. All images were acquired using a Nikon Eclipse 50i microscope system and Image-Pro Plus 6.0 software with standard image processing. The percentage of apoptosis was calculated by the percentage of TUNEL-positive nuclei/percentage of DAPI-stained nuclei.

### Western blot

RIPA buffer (Beyotime Biotechnology, China) was used to extract total protein from the tumor tissue, and the BCA method was used for quantitative detection of protein. The proteins were isolated in 12% sodium dodecyl sulfate–polyacrylamide gel electrophoresis (SDS-PAGE) and transferred onto a polyvinylidene fluoride (PVDF) membrane. After blocking with 5% skim milk at room temperature, the PVDF membrane was incubated with the specific primary antibodies against PGC-1α, PINK1, Parkin (ABclonal Technology, China; rabbit polyclonal antibody, 1:1000), Bax, Bcl-2, caspase-9, caspase-3, and cleaved caspase-3 (Cell Signaling Technology, USA; rabbit anti-mouse monoclonal antibody, 1:1000), at 4 °C overnight. After incubation with the secondary antibody (Cell Signaling Technology, USA; horseradish peroxidase [HRP]-conjugated goat anti-rabbit immunoglobulin G [IgG], 1:2000), the protein bands were visualized by enhanced chemiluminescence reagent (Merck Millipore, USA) and detected using a bioimaging system (Bio-Rad ChemiDoc™ XRS+ Imaging System, USA). The results were quantified by ImageJ software. The relative protein expression was obtained by the gray value of target protein/GAPDH.

### Morphological observation by transmission electron microscopy (TEM)

The tumor tissue samples were harvested and fixed at 4 °C for 2–4 h with glutaraldehyde. They were then fixed at room temperature (20 °C) for 2 h with 1% osmium and 0.1 M phosphate buffer (PB) and rinsed with 0.1 M PB. After dehydration in graded ethanol and acetone, the samples were embedded in Epon 812. The ultrathin sections were stained with uranium acetate and lead citrate then observed by TEM (Hitachi, Japan).

### Statistical analysis

All data were verified for normality in each experiment, and the unpaired two-tailed Student *t*-test was used to analyze the differences between groups. Statistical analyses were performed with GraphPad Prism software (version 8). All assays were performed at least three times, and the data were expressed as the mean ± standard error of the mean (SEM). A *P*-value less than 0.05 was considered statistically significant.

## Results

### Inhibition of colon cancer growth by *Plasmodium* infection in mice

**Fig. 1:**
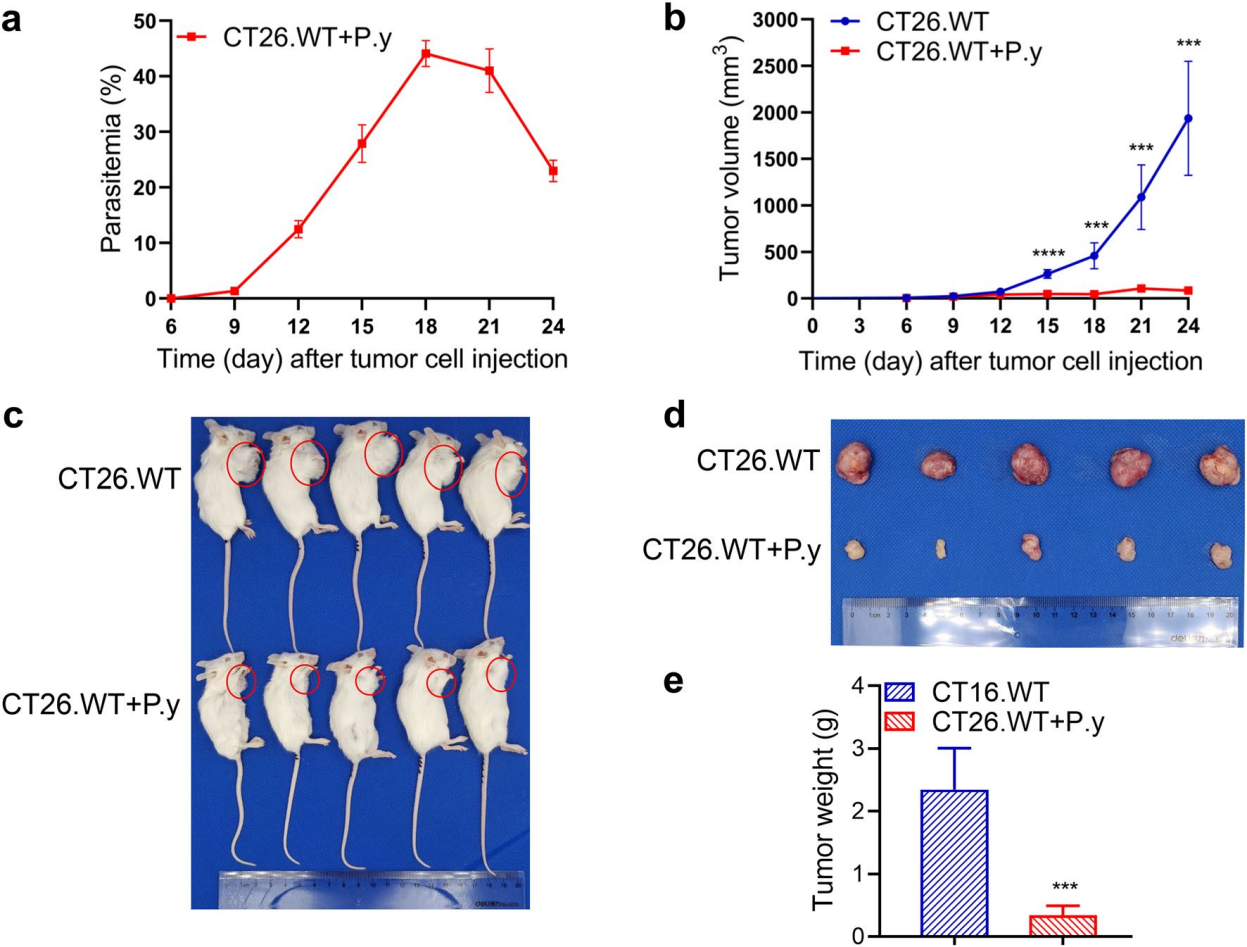
*Plasmodium yoelii* infection suppressed tumor growth in a murine colon cancer model. BALB/c mice were subcutaneously injected with CT26.WT cells under the right forelimb. On the sixth day, the tumor-bearing mice were intraperitoneally injected with either *P. yoelii*-infected erythrocytes or normal erythrocytes. **a** Parasitemia of the CT26.WT + P.y group. **b** Tumor volume was measured over time from the day of tumor cell inoculation. Day 15 (*t*-test, *t*_(8)_ = 10.01, *P* < 0.0001); day 18 (*t*-test, *t*_(8)_ = 6.515, *P* = 0.0002); day 21 (*t*-test, *t*_(8)_ = 6.290, *P* = 0.0002); day 24 (*t*-test, *t*_(8)_ = 6.730, *P* = 0.0001). **c**, **d** The mice were euthanized on day 24, and tumors were harvested for weighing, photographing, and further analysis. **e** Weight of the tumors (*t*-test, *t*_(8)_ = 6.618, *P* = 0.0002). CT26.WT denotes the control group and CT26.WT + P.y denotes the *P. yoelii*-infected group. The results are shown as mean ± SEM (*n* = 5). ***P < 0.001, ****P < 0.0001

The mouse model of colon cancer was established by subcutaneous inoculation. On the sixth day after tumor cell injection, erythrocytes infected with *P. yoelii* were injected intraperitoneally into tumor-bearing mice, and parasitemia was monitored dynamically. There was a peak period following *Plasmodium* infection, after which the levels of parasitemia gradually decreased (Fig. 1a). There was no significant difference in tumor size between the *Plasmodium* infection group (3.487 ± 0.758 mm^3^) and the control group (5.905 ± 1.357 mm^3^) on the sixth day. From the 15th day after tumor cell inoculation, not only was the tumor size in the *P. yoelii*-infected group significantly reduced compared to the control group, but tumor growth was markedly slower (Fig. 1b). On the 24th day after tumor cell inoculation, the tumors of the *P. yoelii*-infected group were strikingly smaller in size and lower in weight than those in the control group (Fig. 1c–e). The results indicate that *Plasmodium* infection can inhibit the growth of colon cancer in mice.

### *Plasmodium* infection suppressed the proliferation of colon cancer cells in mice

**Fig. 2:**
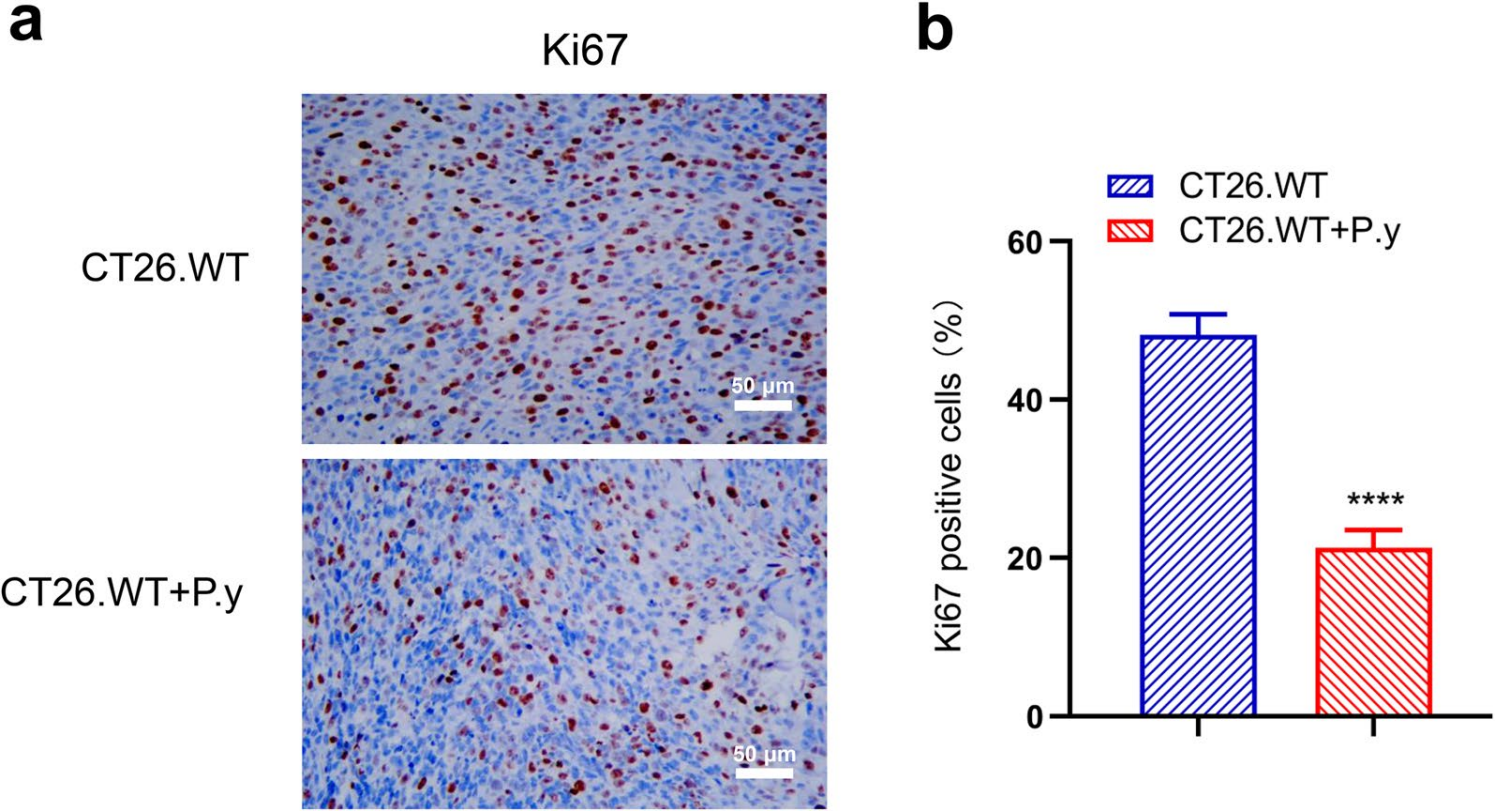
*Plasmodium* infection inhibited the proliferation of tumor cells in colon cancer tissues. **a** Immunohistochemical staining demonstrates that the Ki67-positive cells were decreased in the CT26.WT + P.y group (×400). **b** The percentage of Ki67 expression in tumor tissues of the two groups (*t*-test, *t*_(8)_ = 17.51, *P* < 0.0001). Brown areas represent positive expression. Scale bar = 50 µm. The results are shown as mean ± SEM (*n* = 5). ****P < 0.0001

To determine the effect of *Plasmodium* infection on the proliferation of colon cancer cells in mice, we examined the expression of Ki67, a cell proliferation marker, using immunohistochemical staining. We found that the expression of Ki67 was obviously decreased in the *P. yoelii* infection group compared with the control group (Fig. 2a). There were statistically significant differences in the percentage of Ki67-positive cells between the two groups (Fig. 2b). The results suggest that *Plasmodium* infection can suppress the proliferation of colon cancer cells in vivo.

### Apoptosis of colon cancer cells induced by *Plasmodium* infection in mice

**Fig. 3:**
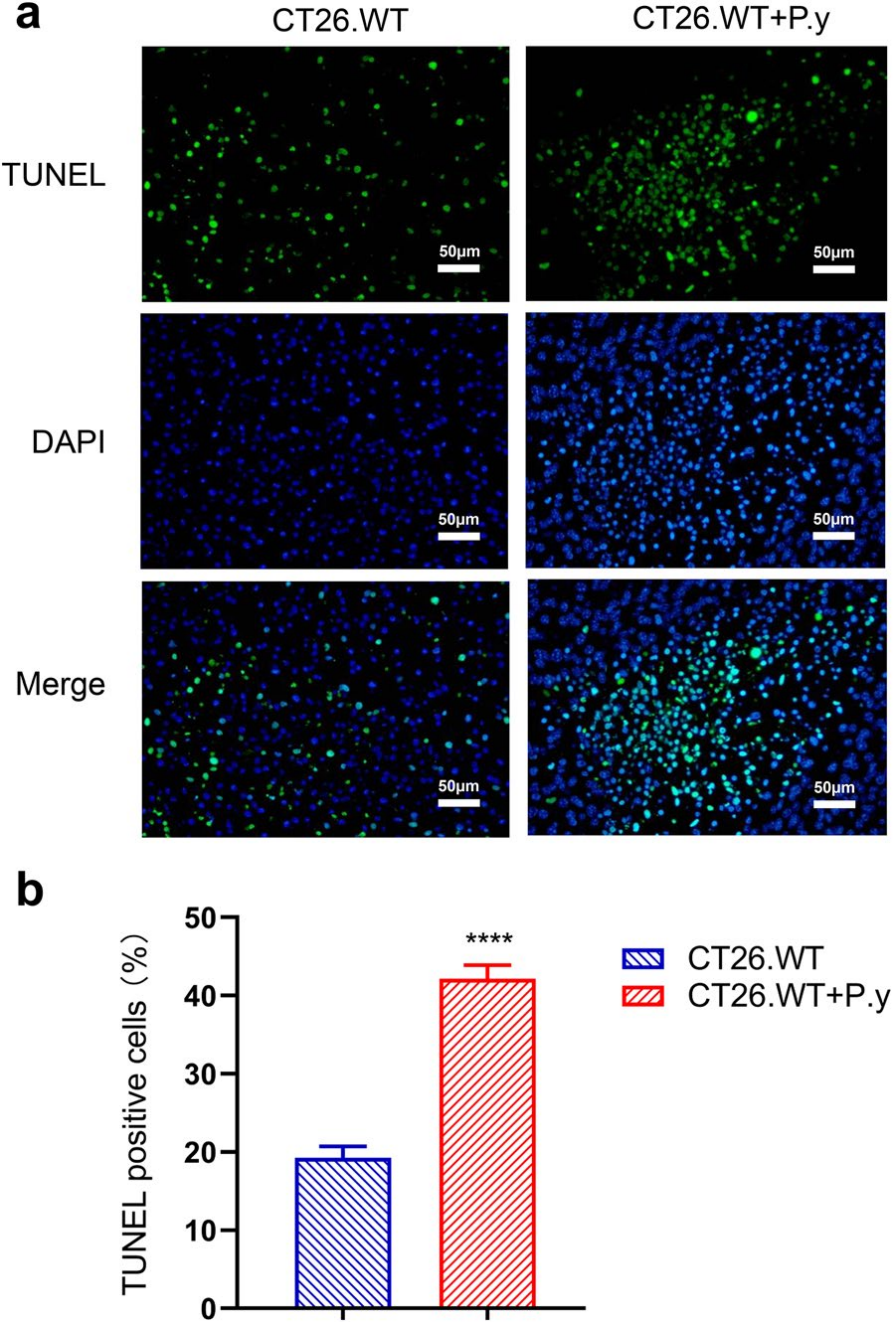
Apoptosis in colon cancer induction by *Plasmodium* infection. **a** Apoptosis was observed under a fluorescence microscope (×400). **b** Quantitative estimation of the proportion of apoptotic cells in each group (*t*-test, *t*_(8)_ = 22.31, *P* < 0.0001). Green fluorescence indicates the TUNEL-positive nuclei, blue represents DAPI stained nuclei, and a blend indicates apoptotic cells. The results are shown as mean ± SEM (*n* = 5). Scale bar = 50 µm. **** *P* < 0.0001

Apoptosis of tumor cells was detected by TUNEL assay, in which enhanced green fluorescence represented the TUNEL-positive cells. Compared to the control group, a large number of TUNEL-positive cells was detected in the *P. yoelii*-infected group (Fig. 3a). The CT26.WT + P.y group displayed a higher proportion of cell apoptosis than the CT26.WT group (Fig. 3b). These results indicate that *Plasmodium* infection can induce apoptosis of colon cancer cells.

### The mitochondrial apoptosis activation by *Plasmodium* infection

**Fig. 4:**
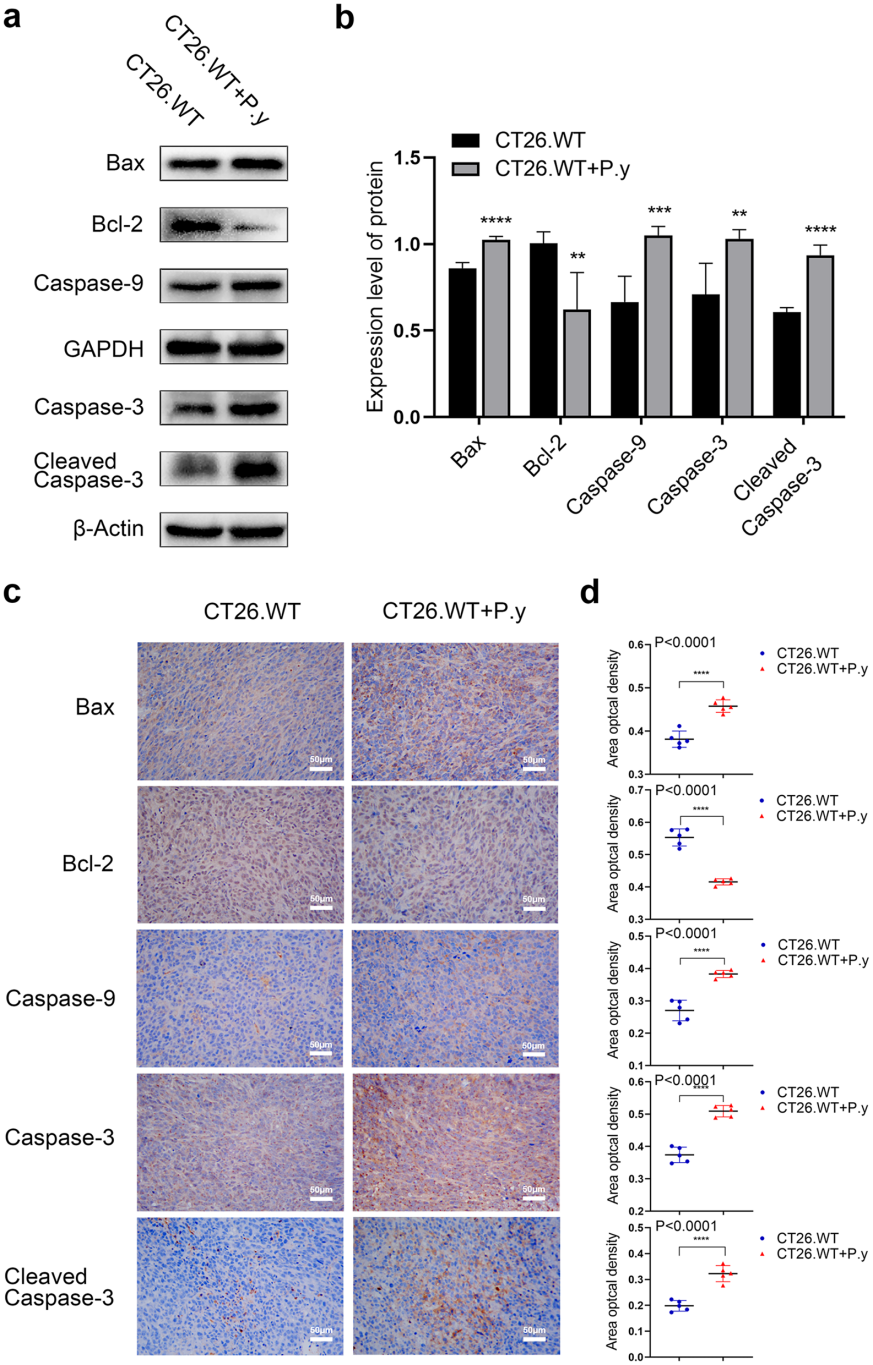
The mitochondrial pathway regulation by *Plasmodium* infection in colon cancer-bearing mice. **a** The expression of mitochondria-mediated apoptosis proteins (Bax, Bcl-2, caspase-9, caspase-3 and cleaved caspase-3) was analyzed by western blot. **b** Quantification of relative intensity of western blot signals: Bax (*t*-test, *t*_(8)_ = 9.438, *P* < 0.0001); Bcl-2 (*t*-test, *t*_(8)_ = 3.822, *P* = 0.0051); caspase-9 (*t*-test, *t*_(8)_ = 5.462, *P* = 0.0006); caspase-3 (*t*-test, *t*_(8)_ = 3.836, *P* = 0.0050); cleaved caspase-3 (*t*-test, *t*_(8)_ = 11.36, *P* < 0.0001). **c**, **d** Immunohistochemical staining and quantification of apoptosis protein expression in the mitochondrial pathway. Bax (*t*-test, *t*_(8)_ = 7.222, *P* < 0.0001); Bcl-2 (*t*-test, *t*_(8)_ = 10.92, *P* < 0.0001); caspase-9 (*t*-test, *t*_(8)_ = 7.465, *P* < 0.0001); caspase-3 (*t*-test, *t*_(8)_ = 10.23, *P* < 0.0001); cleaved caspase-3 (*t*-test, *t*_(8)_ = 7.458, *P* < 0.0001). The results are presented as mean ± SEM (*n* = 5). Scale bar = 50 µm. ** *P* < 0.01, *** *P* < 0.001, **** *P* < 0.0001

To further explore the mechanism of *Plasmodium* infection-induced apoptosis in colon cancer cells, we used western blot to detect the expression of apoptosis-related proteins in the mitochondrial pathway. Results showed that the expression of proapoptotic proteins, including Bax, caspase-9, and cleaved caspase-3, were upregulated, while the expression of the antiapoptotic protein Bcl-2 was downregulated after *Plasmodium* infection compared with the control group (Fig. 4a, b).

In addition, immunohistochemical staining was used to observe the effects of *Plasmodium* infection on the expression of proteins in the mitochondria-mediated apoptosis pathway (Fig. 4c, d). Consistent with the results of western blot, we found that the expression of Bax, caspase-9, and cleaved caspase-3 were noticeably increased in the *P. yoelii*-infected model, whereas Bcl-2 expression was lower in the *P. yoelii*-infected group than in the control group. These results suggest that *Plasmodium* infection was involved in regulating the expression of apoptosis-related proteins in the mitochondrial pathway leading to mitochondrial apoptosis.

### The mitochondria and nuclei of colon cancer cells were altered by *Plasmodium* infection in tumor-bearing mice

**Fig. 5:**
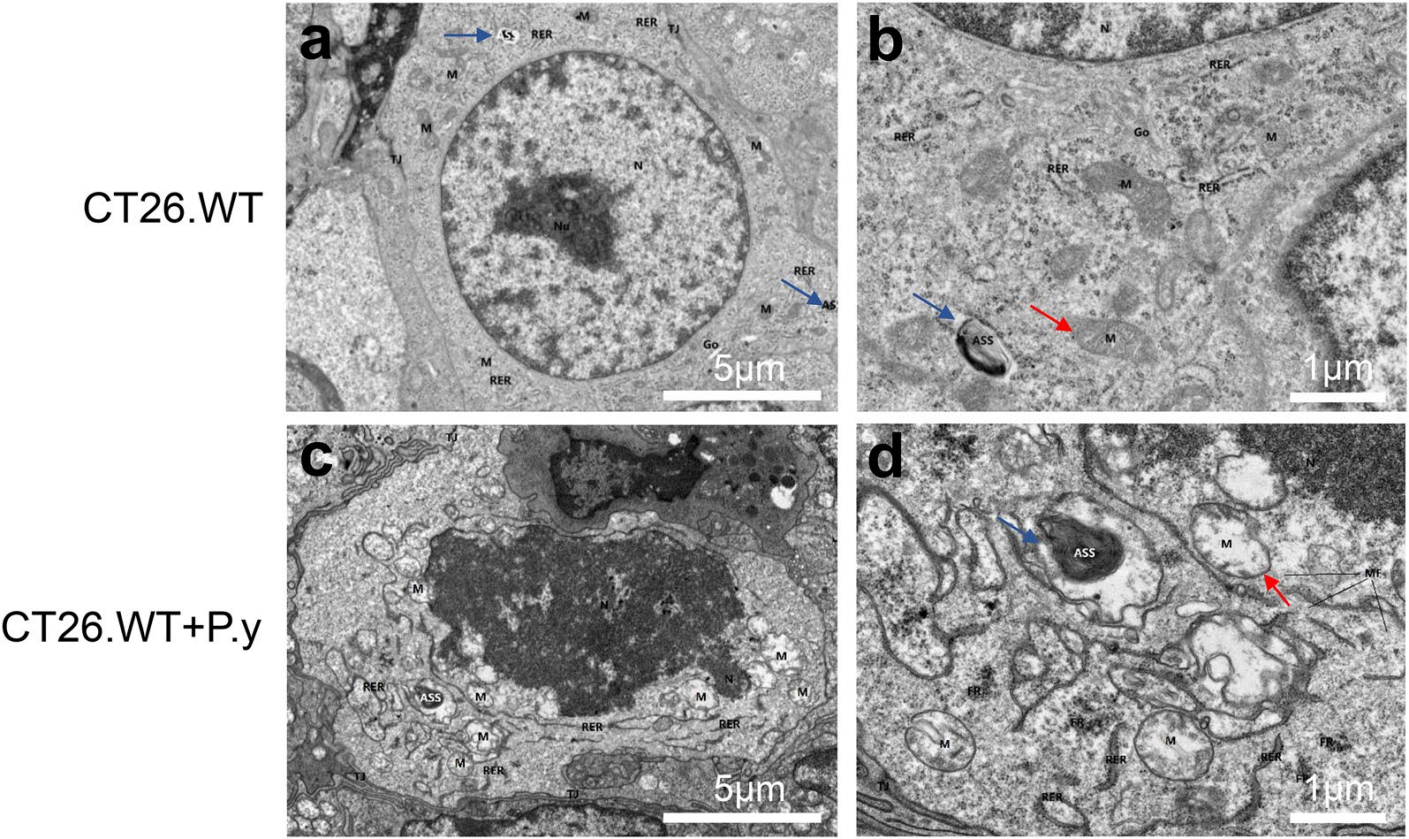
Changes in tumor cell ultrastructure after *Plasmodium* infection. The ultrastructural changes in tumor cells in the two groups were observed by TEM. The damaged cell nuclei and mitochondria were observed in the CT26.WT + P.y group. The mitochondria (M, red arrow) were swollen and the cristae disappeared and vacuolated, with chromatin condensation and nuclear disintegration (N: nucleus). The number of autolysosomes (ASS, blue arrow) in the CT26.WT + P.y group was more than that in the CT26.WT group. **a**, **c** The magnification is ×2000, scale bar = 5 µm. **b**, **d** The magnification is ×6000, scale bar = 1 µm

As shown in Fig. 5, we used TEM to further observe changes in the ultrastructure of colon cancer cells among the two groups. After *Plasmodium* infection, colon cancer cells displayed severe edema, nuclear atypia, chromatin condensation, and nuclear disintegration. Mitochondria were severely swollen and enlarged, the matrix in the membrane dissolved, and the cristae disappeared and vacuolated in the CT26.WT + P.y group compared to the control group. Moreover, under TEM, the number of autolysosomes in the CT26.WT + P.y group was less than in the control group. The results reveal that *Plasmodium* infection-induced tumor cells damage was associated with mitochondrial and nuclear damage in colon cancer cells.

### *Plasmodium* infection disrupted mitochondrial biogenesis in the colon cancer model

**Fig. 6:**
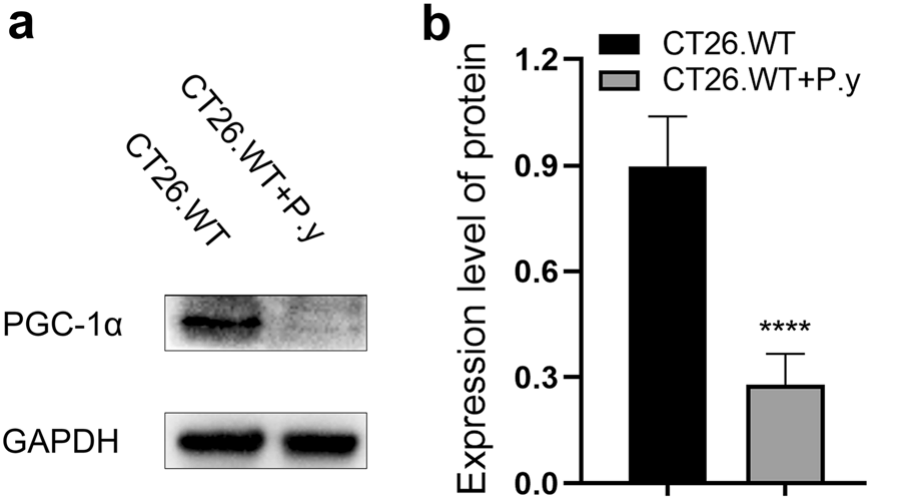
*Plasmodium* infection inhibited mitochondrial biogenesis in colon cancer. **a** Western blot analysis showed that the expression of the PGC-1α protein was decreased in the CT26.WT + P.y group; GAPDH was used as a loading control. **b** The density ratio of PGC-1α/GAPDH was decreased in the CT26.WT + P.y group compared to the control group (*t*-test, *t*_(8)_ = 8.356, *P* < 0.0001). The results are shown as the density mean ± SEM (*n* = 5). **** *P* < 0.0001

To assess the effect of *P. yoelii* infection on mitochondrial biogenesis, we used western blot to analyze a key regulator of mitochondrial biogenesis, PGC-1α. The result showed that the expression of the PGC-1α protein in the *P. yoelii*-infected group was evidently reduced in contrast to the control group (Fig. 6). The results demonstrate that *Plasmodium* infection could disrupt mitochondrial biogenesis in colon cancer.

### *Plasmodium* infection inhibited mitophagy in colon cancer-bearing mice 

**Fig. 7:**
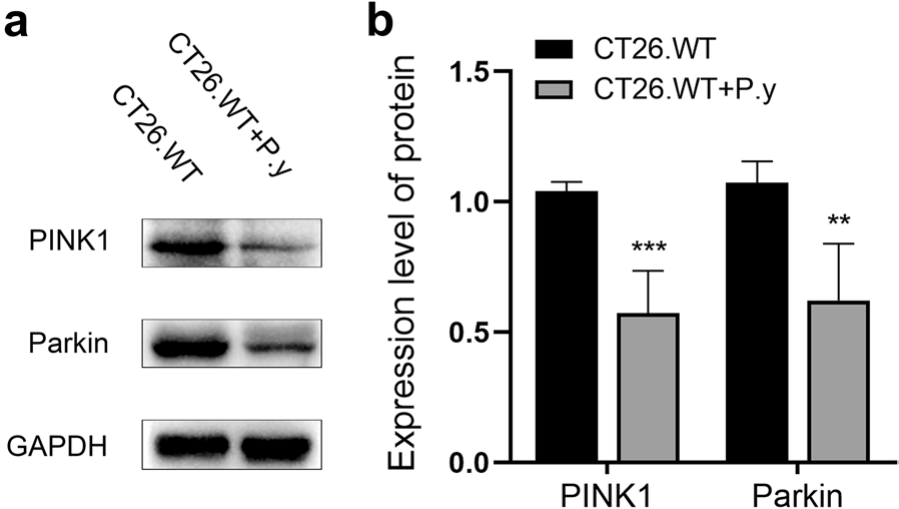
*Plasmodium* infection inhibited mitophagy in the murine colon cancer model. **a** The western blot results showed that the expression of PINK1 and Parkin were both decreased in the CT26.WT + P.y group. **b** Quantification of protein expression was normalized to GAPDH: PINK1 (*t*-test, *t*_(8)_ = 6.558, *P* < 0.0002); Parkin (*t*-test, *t*_(8)_ = 4.758, *P* = 0.0014). The results are shown as the density mean ± SEM (*n* = 5). **P < 0.01, ***P < 0.001

The PINK1/Parkin-mediated pathway is the most important pathway for mitophagy, which is crucial for maintaining mitochondrial function and integrity [32]. To evaluate the effect of *Plasmodium* infection on mitophagy in colon cancer cells, we therefore examined the expression of these two mitophagy-related proteins. Western blot results showed that the levels of PINK1 and Parkin were decreased after *Plasmodium* infection (Fig. 7). The results suggest that *Plasmodium* infection can inhibit mitophagy, leading to mitochondrial dysfunction in colon cancer.

## Discussion

As one of the most common malignant tumors, colon cancer poses an extreme threat to human health [33]. Because of the high incidence and mortality associated with colon cancer, researchers devoted to reducing mortality and improving patients' quality of life are continuously seeking new treatments to inhibit the growth, recurrence, and metastasis of colon cancer [34]. The murine model is a common model for cancer research [35], and the *P. yoelii* 17XNL nonlethal strain of malaria parasite can be used to infect the mice for examining its antitumor effect without causing the death of the animals [5–7]. Previously, our team and other researchers have all found that *Plasmodium* infection had antitumor effects on some cancers in different mouse models, but it was not clear whether it would have similar influences on colon cancer. Therefore, in this study, we investigated the anti-colon cancer effect of *Plasmodium* infection in mice.

We found that *Plasmodium* infection slowed the growth of tumors and reduced the size and weight of the tumors in the murine colon cancer model. These results indicate that *Plasmodium* infection could play an anti-colon cancer role in mice.

Infinite proliferation is one of the important characteristics of cancer cells [36]. Inhibition of cell proliferation can effectively inhibit tumor growth [37]. The Ki67 protein, a nuclear antigen related to cell proliferation, is expressed in G1, G2, S, and M phases, but no expression in the G0 phase [38]. As one of the most reliable indicators for detecting cell proliferation activity of tumor cells, the function of the Ki67 protein is closely associated with the process of cell mitosis [39, 40]. In this study, the expression of Ki67 in colon cancer tissues was distinctly decreased after parasite infection, suggesting that *Plasmodium* infection could suppress the proliferation of colon cancer cells and further inhibit tumor growth in tumor-bearing mice. When the mice were euthanized on day 24 after tumor inoculation, the parasitemia was still higher than 20%. It is unclear whether a long- or short-term tumor inhibitory effect would persist after clearance of parasitemia in our model, but it is worth investigating in the future, especially the mechanisms behind the anti-colon cancer effect.

Apoptosis, the most common form of programmed cell death, is one of the keys to maintaining healthy cell homeostasis [41]. Resistance to apoptosis is another crucial characteristic of tumor cells [42]. Induction of apoptosis is one of the important approaches in antitumor drug research [43]. There are three pathways of apoptosis in mammalian cells, including the mitochondrial pathway, the death receptor pathway, and the endoplasmic reticulum stress pathway [44–46]. Mitochondrial apoptosis is the primary form of apoptosis [47]. When mitochondria are damaged, Bax is transported into mitochondria to initiate apoptosis, triggering cytochrome *c* release in the mitochondria, which further activates the caspase cascade resulting in mitochondrial apoptosis [48–50].

In the tumor-bearing mice infected with *P. yoelii*, we found an increased proportion of TUNEL-positive cells, upregulated expression of proapoptotic factors including Bax, caspase-9, and cleaved caspase-3, and downregulated expression of antiapoptotic factor Bcl-2 in colon cancer tissues, indicating that *Plasmodium* infection promoted mitochondria-mediated apoptosis. Since resistance to apoptosis is one of the typical characteristics of cancer, the induction of apoptosis leads to the death of cancer cells. Thus, it is reasonable to assume that *Plasmodium* infection exerts an anti-colon cancer effect by inhibiting tumor growth through induction of apoptosis. This anti-colon cancer effect of *Plasmodium* infection is similar to the effect of some antitumor drugs that eliminate cancer cells by inhibiting proliferation and inducing apoptosis [51, 52].

Mitochondrion, a subcellular organelle which plays a major role in cell energy control and metabolism, is essential for the survival and growth of cells [53, 54]. As an important regulator involved in the pathways of mitochondrial biogenesis, mitophagy, carcinogenesis, and tumor cell death, including mitochondria-mediated apoptosis, mitochondria have been considered as potential therapeutic targets for cancer [55]. In this study, we found that the mitochondria and nuclei of colon cancer cells were severely damaged in *Plasmodium*-infected mice, mainly with the mitochondrial cristae disappearance and vacuolation. The results suggest that the antitumor effect of *Plasmodium* infection might relate to mitochondria. The maintenance of mitochondrial homeostasis depends on the interaction between mitochondrial biogenesis and mitophagy [56]. PGC-1α protein dominates mitochondrial biogenesis and protects tumor cells from apoptosis [57, 58]. Previous studies both in vivo and in vitro have confirmed that downregulated expression of PGC-1α induced apoptosis through the mitochondrial pathway [59]. A recent study revealed a potential relationship between mitochondrial biogenesis and apoptosis [60]. In the early stages of apoptosis, mitochondria produce energy to maintain homeostasis, leading to enhanced mitochondrial biogenesis [61]. As the massive production of reactive oxygen species (ROS), mitochondrial energy metabolism is unbalanced, mitochondrial biogenesis is weakened, mitochondrial membrane potential is decreased, cytochrome *c* is released, and the caspase pathway is activated, leading to apoptosis [62, 63]. In this study, we found that the expression of PGC-1α protein in colon cancer cells was reduced after *P. yoelii* infection. The results demonstrate that *Plasmodium* infection inhibited mitochondrial biogenesis in colon cancer cells. Inhibition of mitochondrial biogenesis promoted apoptosis and inhibited proliferation, which may be one of the mechanisms underlying the antitumor effect of *Plasmodium* infection.

Autophagy is a process in which intracellular components are degraded into autophagosomes and combined with the lysosomes to form autolysosomes, resulting in the clearance of damaged organelles [64, 65]. Studies have increasingly demonstrated that autophagy is a self-protective mechanism for cells [66, 67]. Mitophagy is mitochondria-specific autophagy, a self-protective process in which dysfunctional mitochondria are selectively degraded [68]. When mitochondria are damaged, PINK1 protein hydrolysis is restrained and it is steadily expressed on the mitochondrial outer membrane. Then, PINK1 recruits Parkin to the outer membrane of mitochondria and ubiquitinates multiple mitochondrial outer membrane proteins to mediate mitophagy, eventually removing damaged mitochondria [69, 70]. In the study, we found that the number of autolysosomes was decreased and the expression of PINK1 and Parkin proteins was decreased in colon cancer cells after *Plasmodium* infection. The results indicate that *Plasmodium* infection inhibited mitophagy in colon cancer-bearing mice, leading to mitochondrial damage and dysfunction. Many studies have shown that suppression of autophagy, including mitophagy, accelerates apoptosis, resulting in tumor cell death and ultimately inhibiting tumor growth [71]. Therefore, in colon cancer cells, *Plasmodium* infection inhibited mitophagy, thus promoting apoptosis and ultimately inhibiting tumor growth, which may be one of the anti-colon cancer mechanisms of *Plasmodium* infection.

**Fig. 8:**
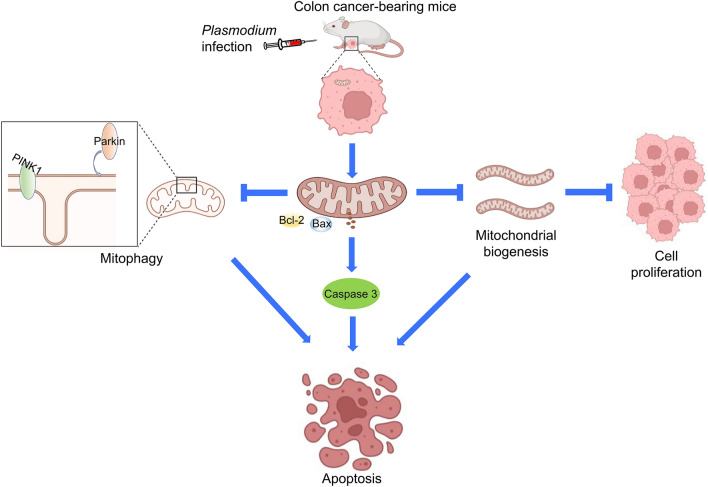
The anti-colon cancer mechanisms of *Plasmodium* infection in vivo. In the murine colon cancer model, *Plasmodium* infection inhibits the proliferation of tumor cells and induces mitochondrial apoptosis. Furthermore, *Plasmodium* infection disturbs mitochondrial biogenesis, leading to the inhibition of proliferation and the promotion of apoptosis. Mitophagy is inhibited by *Plasmodium* infection, contributing to mitochondrial dysfunction. *Plasmodium* infection inhibits the growth of tumors to exert the tumor suppression function

In this study, although we found that *Plasmodium* infection could inhibit colon cancer cell proliferation and induce apoptosis by controlling mitochondrial biogenesis and mitophagy (Fig. 8), it is not clear exactly what is behind this. The suppression function may be connected with the subsequent effect of cytokines induced by immune effects after *Plasmodium* infection, the impact of the components or metabolites of *Plasmodium* itself, or the result of changes in non-coding RNA expression caused by *Plasmodium* infection. The mechanism of *Plasmodium* infection controlling mitochondrial biogenesis and mitophagy remains to be further explored.

## Conclusions

In this study, our findings firstly demonstrate that *P. yoelii* 17XNL infection had significant anti-colon cancer effects in mice by inhibiting tumor cell proliferation and promoting mitochondrial apoptosis, which may relate to the inhibition of mitochondrial biogenesis and mitophagy. The results presented here may inspire a new approach for the treatment of colon cancer.

## Data Availability

The datasets supporting the findings of this article are included within the article and its additional file.

## Abbreviations

*P. yoelii*: *Plasmodium yoelii*
RPMI 1640: Roswell Park Memorial Institute 1640 medium
FBS: Fetal bovine serum
TUNEL: Terminal deoxynucleotidyl transferase (TdT) dUTP nick-end labeling
TEM: Transmission electron microscopy
SDS-PAGE: Sodium dodecyl sulfate–polyacrylamide gel electrophoresis
SEM: Standard error of the mean
